# PhyloPGM: boosting regulatory function prediction accuracy using evolutionary information

**DOI:** 10.1093/bioinformatics/btac259

**Published:** 2022-06-27

**Authors:** Faizy Ahsan, Zichao Yan, Doina Precup, Mathieu Blanchette

**Affiliations:** School of Computer Science, McGill University, Montreal H3A 0G4, Canada; School of Computer Science, McGill University, Montreal H3A 0G4, Canada; School of Computer Science, McGill University, Montreal H3A 0G4, Canada; School of Computer Science, McGill University, Montreal H3A 0G4, Canada

## Abstract

**Motivation:**

The computational prediction of regulatory function associated with a genomic sequence is of utter importance in -omics study, which facilitates our understanding of the underlying mechanisms underpinning the vast gene regulatory network. Prominent examples in this area include the binding prediction of transcription factors in DNA regulatory regions, and predicting RNA–protein interaction in the context of post-transcriptional gene expression. However, existing computational methods have suffered from high false-positive rates and have seldom used any evolutionary information, despite the vast amount of available orthologous data across multitudes of extant and ancestral genomes, which readily present an opportunity to improve the accuracy of existing computational methods.

**Results:**

In this study, we present a novel probabilistic approach called PhyloPGM that leverages previously trained TFBS or RNA–RBP binding predictors by aggregating their predictions from various orthologous regions, in order to boost the overall prediction accuracy on human sequences. Throughout our experiments, PhyloPGM has shown significant improvement over baselines such as the sequence-based RNA–RBP binding predictor RNATracker and the sequence-based TFBS predictor that is known as FactorNet. PhyloPGM is simple in principle, easy to implement and yet, yields impressive results.

**Availability and implementation:**

The PhyloPGM package is available at https://github.com/BlanchetteLab/PhyloPGM

**Supplementary information:**

Supplementary data are available at *Bioinformatics* online.

## 1 Introduction

The sequence and cell-type-specific interaction between proteins and DNA/RNA is the main effector that drives both transcriptional and post-transcriptional regulation (Stefl *et al.*, 2005). For DNA-protein interaction, the binding of transcription factors (TFs) to specific DNA regions plays a crucial role in the gene regulatory network, which is influenced by diverse factors including the presence of motifs that are specific 6–20 bps patterns in DNA sequences, cell-type-specific context such as DNA accessibility and methylation, and the presence of other bound TFs (Slattery *et al.*, 2014). For RNA–protein interaction, representative examples include RNA splicing that prepares nascent RNA transcripts for maturation and the subsequent localization which transports the messenger RNA (mRNA) to certain subcellular compartments where their products are needed. These regulatory processes are mediated by a diverse population of trans-acting protein agents, each having an affinity for a specific DNA/RNA motif, and aberrations from their usual interaction scheme are known to implicate a series of neurological disorders and possibly cancer (Lukong *et al.*, 2008). Therefore, it is crucial to characterize the TF and the RBP binding specificity in order to comprehend the gene regulatory network, to scrutinize the associated disease pathways and possibly, to develop related therapeutic approaches.

Dedicated wet-lab protocols have been developed over the years in order to reveal the interaction between the trans-acting protein agents and their corresponding cis-acting sequence elements. Chromatin immunoprecipitation sequencing (ChIP-Seq) experiment is one such example that identifies binding sites of one transcription factor in one cell type *in vivo* within a resolution of ∼200 bps (Johnson *et al.*, 2007). The ENCODE consortium has produced ChIP-Seq experiments data for hundreds of transcription factors in dozens of cell types (Consortium *et al.*, 2012). Similarly, CLIP-Seq (Cross-Linking Immunoprecipitation and high throughput RNA Sequencing) experiments and its variants such as PARCLIP (Hafner *et al.*, 2010), HITSCLIP (Licatalosi *et al.*, 2008) and ICLIP (Konig *et al.*, 2010) can identify *in vivo* RNA binding to a given RBP. In the CLIP-Seq experiment, RBP and RNA are cross-linked with UV light, which is followed by lysing, immunoprecipitation and sequencing. Although CLIP-Seq experiments yield a resolution of ∼100 bps in RNAs that are bound with an RBP, the exact location of binding is unknown, which is also the case in ChIP-Seq experiments. Moreover, it is impractical to conduct ChIP-Seq and CLIP-Seq experiments exhaustively for each TFs, RBPs and cell types combination. Therefore, a computational method that predicts the TFBSs and RNA–RBP interaction is required in order to profile the sequence specificity of TFs and RBPs.

Recent computational methods to predict TFBS and RNA–RBP binding are heavily dominated by deep learning-based approaches in terms of prediction accuracy, e.g., convolutional neural networks (Alipanahi *et al.*, 2015) and a hybrid of convolutional neural network and recurrent neural network (Pan *et al.*, 2018). In general, a DNA sequence of roughly 1000 bps or an RNA sequence of ∼100 bps is represented as a one-hot encoded vector, which is then passed through the deep neural network of choice to predict whether the DNA sequence will be bound by the TF or the RNA sequence will interact with the RBP of interest. Although, the deep learning approaches (Pan and Shen, 2017, 2018, Quang and Xie, 2016; Zhang *et al.*, 2016) have outperformed the classical computational methods and shallow machine learning approaches (Fukunaga *et al.*, 2014; Hiller *et al.*, 2006; Kazan *et al.*, 2010; Li *et al.*, 2010; Maticzka *et al.*, 2014; Pietrosanto *et al.*, 2016), they are often prone to high false-positive rate and are yet to be established as wet-lab alternatives.

Due to the biological importance associated with gene regulatory events, patterns latent in the DNA and RNA sequence that allow binding to TFs and RBPs are thought to be highly conserved during evolution. Indeed, the conservation of regulatory functions across the multitude of orthologous regions in different organisms has been observed according to the orthologs conjecture (Chen and Zhang, 2012; Cooper and Brown, 2008; Papatsenko *et al.*, 2006; Shabalina *et al.*, 2004; Shiraishi *et al.*, 2001; Stamboulian *et al.*, 2020). Intuitively, the sequence function conservation-based approaches should yield better models. However, such approaches may suffer from binding sites turnover phenomenon, where the number of binding sites in a given region is maintained despite the sequence itself is not conserved in the orthologous regions (Moses *et al.*, 2006; Sinha and Siggia, 2005). Therefore, a more sophisticated operation is required to use the sequence function conservation property rather than the crude combination of sequence conservation score with the deep learning methods (Ahsan *et al.*, 2020).

**Fig. 1.**
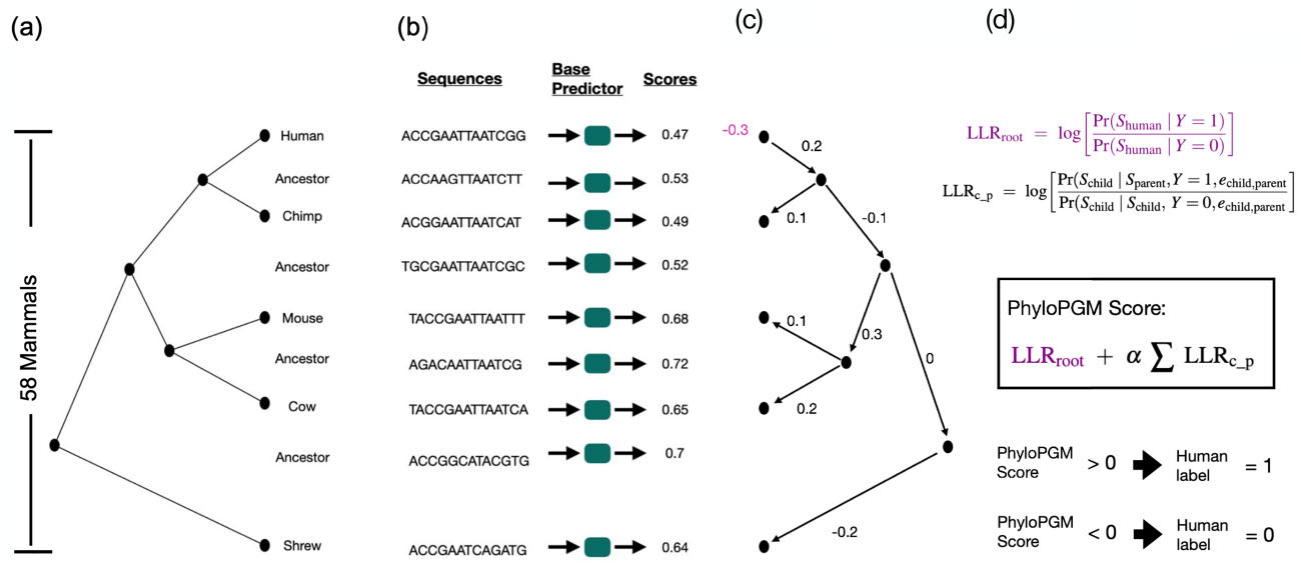
PhyloPGM workflow. (**a**) the phylogenetic tree, (**b**) the input human sequence and its orthologs are fed to trained base predictor in order to obtain the orthologous prediction scores, (**c**) each branch weight denotes the log likelihood ratio of child score given parent score (LLR${}_{c\_p}$) is obtained by treating human as the root species and the human weight denotes the log likelihood ratio of root (LLR${}_{\text{root}}$), (**d**) equations used to compute the log likelihood ratio and the PhyloPGM score. $e_{\text{child},\text{parent}}$ is the evolutionary distance between child and parent species. The human sequence label is assigned 1 (binding site) if the PhyloPGM score > 0, otherwise 0 (non-binding site)

In this study, we present an aggregation approach called PhyloPGM, which aims to boost the accuracy of a pre-trained base predictor for a specific type of regulatory function (see Fig. 1). The base predictor is a machine learning model that assigns a prediction score (real number) to a given input sequence. In this article, we use PhyloPGM for two types of functional prediction tasks: transcription factor binding prediction [using FactorNet in Quang and Xie (2019) as base predictor] and RBP binding prediction [using RNATracker in Yan *et al.* (2019)]. To obtain a prediction on a given human sequence, the base predictor is first applied to that sequence and its orthologous regions from up to 58 other mammalian species as well as up to 57 computationally reconstructed ancestral sequences. PhyloPGM then aggregates the prediction scores using a phylogenetically informed probabilistic graphical model, essentially computing a likelihood ratio test that contrasts the two hypotheses that the human sequence is either a positive (Y = 1) example or a negative (Y = 0) example.

PhyloPGM takes advantage of the fact that selective pressure rarely induces changes to crucial functions encoded in the regulatory regions. Hence, predictions made on orthologous and ancestral sequences should be consistent and informative about the function of the given human sequence. Particularly, in cases where the base predictor is inaccurate and in regulatory regions where changes to the function are relatively rare, PhyloPGM could in principle use predictions made on orthologous/ancestral sequences to correct the prediction made on the human sequence. More importantly, since PhyloPGM treats the base predictor as a black box (i.e. it does not need any information about the base predictor’s inner workings), the tool is highly flexible and applicable to a wide variety of sequence-function prediction methods for which the community has developed so far.

To put PhyloPGM into a broader context, since the goal of PhyloPGM is to combine prediction scores on a set of related orthologous and ancestral sequences, PhyloPGM can be considered as an instantiation of multi-instance learning (MIL), a class of ML approaches that classify a group of related instances, termed as a bag (Dietterich *et al.*, 1997). The MIL classifier labels the bag as positive if at least one of the instances is positive, otherwise negative. The classical MIL algorithms assume instances to be i.i.d., though there are MIL algorithms that handle non-i.i.d. cases as well (Ping *et al.*, 2010; Zhou *et al.*, 2009). Gao and Ruan (2015) used MIL with DNA structure data for *in vitro* TFBSs predictions in the mouse without phylogenetic context. The MIL algorithms are extensively reviewed in Amores (2013) and Foulds and Frank (2010). Our requirement for the aggregating approach differs from the classical MIL in three principal ways: (1) the instances are prediction scores obtained from a base predictor (rather than raw sequences or feature vectors); (2) the goal is to predict the label of a specific example from each bag (corresponding to the human sequence); (3) instances in the bag are not i.i.d. but are phylogenetically related through a known and fixed tree.

## 2 Methods

We define the problem of aggregating prediction scores for the purpose of improving prediction accuracy on human sequences as,

**Given**: a set of prediction scores on the orthologous and ancestral genomic sequences obtained from a base model, *B*, which is previously trained using human genomic sequences only, and a phylogenetic tree that relates the involved species.

**Goal**: to predict the label of a human genomic sequence such that the resulting prediction improves the accuracy of *B*.

We first describe the ChIP-Seq data, CLIP-Seq data and orthologous data that are used to demonstrate the efficiency of PhyloPGM. Then, we detail the Factornet and RNATracker models, which are used as base predictors. We conclude by describing the PhyloPGM and PhyloStackNN algorithms.

### 2.1 ChIP-Seq data

A recent DREAM challenge (ENCODE-DREAM in vivo Transcription Factor Binding Site Prediction Challenge) provided ChIP-Seq data from ENCODE for various problems related to TFBSs prediction (Kundaje *et al.*, 2021). One of the challenge’s labeling problems is to build a TFBS prediction model for a given cell type, which provides data consisting of 13 TF/cell-type pairs from 12 TFs and three cell types [liver, PC-3, induced pluripotent stem cell (IPSC)]. The train and test sets both belong to the same cell type. In particular, the test examples come from chromosomes 1, 8 and 21 and examples from the other chromosomes form the training set. The test set contains approximately 8 million examples in total. During the stage of training, we sub-sample negative examples to the same number of positive examples in the train set and keep 20% of the train set separate for validation purposes.

### 2.2 CLIP-Seq data

The RNA–RBP binding dataset is originally curated by Strazar *et al.* (2016), which includes the result of 31 RBP binding experiments conducted under the CLIP-Seq protocol. Each experiment provides 8000 positive examples that contain binding sites for a specific RBP, and 32 000 negative (unbound) examples. Each example is an RNA sequence of 101 nts. A partition of the dataset into a fixed train-test split is followed as in the original paper, with 20% being positive examples in either train or test split. The positive binding sites are identified through several variants of the CLIP-Seq protocol such as PAR-CLIP (Hafner *et al.*, 2010), iCLIP (Konig *et al.*, 2010) and HITS-CLIP (Licatalosi *et al.*, 2008).

### 2.3 Orthologous data

The orthologous regions of each human genomic region in other mammals are extracted using mafsInRegion program (https://hgdownload.soe.ucsc.edu/admin/exe/linux.x86_64/mafsInRegion) from a 100-way vertebrate whole-genome alignment available from the UCSC genome browser (Kent *et al.*, 2002). Only the 58 mammalian sequences were used in this study. The orthologous regions are complemented with computationally predicted ancestral sequences produced by Ancestor1.0 (Diallo *et al.*, 2010). The collected orthologous regions are symmetrically trimmed or joined with surrounding regions to yield sequences sized 1000 bps (TFBS prediction problem) or 101 bps (RBP binding site prediction problem). Each example has on average 80 orthologous and ancestral sequences. We ignore orthologous regions that are smaller than 70% of the corresponding human sequence.

### 2.4 FactorNet as base predictor

FactorNet (Quang and Xie, 2019) is one of the best performing sequence-based model in the recent DREAM competition (Kundaje *et al.*, 2021). In this study, we used the FactorNet architecture that takes sequence information as the only input, which is a genomic sequence and its reverse complement. Each convolution layer in FactorNet contains 32 filters of size 26 and the resulting output is passed through a ReLU activation layer. A dropout layer of *P *=* *0.1 is applied, which is followed by a max-pooling layer with a filter size of 13. Then, a single bidirectional LSTM layer of hidden size 32 is used with a dropout layer of *P *=* *0.5. Afterwards, a fully connected layer of size 128 with ReLU activation function is used. The output of the fully connected layer is then passed through a dropout layer of *P *=* *0.5. The final output layer is a fully connected layer of size 1 with a sigmoid activation function. The mean of the FactorNet outputs from the given genomic sequence and its reverse complement is the final output of the FactorNet. In this study, we trained FactorNet batch-wise (batch size = 128) with early stopping using a validation set.

### 2.5 RNATracker as base predictor

Yan *et al.* (2019) proposed a hybrid of convolutional and recurrent neural network architecture, called RNATracker, to predict the mRNA localization. An mRNA sequence is represented as a one-hot encoded vector, which is then passed through two convolutional layers. Then, a pooling layer is used to aggregate the motif scores. Finally, a bi-directional LSTM with attention is used to aggregate the motif features. The resulting output is passed through a fully connected layer followed by a linear layer to predict the RNA–RBP binding. Although RNATracker was initially developed to predict mRNA localization, the architecture is equally capable of predicting RNA–protein binding. The RNATracker architecture used in this study has two convolutional layers, where each convolution layer has 32 filters of length 10 followed by a max-pooling layer of window size 3 and stride 3. The subsequent bidirectional LSTM layer has 100 hidden units and the following fully connected layer has 128 units. The output layer has one unit with a sigmoid activation function that gives the final prediction score. A dropout layer (*P *=* *0.1) is used after each convolutional and bidirectional LSTM layer.

### 2.6 PhyloPGM: probabilistic aggregation approach

PhyloPGM is a prediction score aggregation approach, which is inspired from the probabilistic graphical models (Koller *et al.*, 2009). Consider a model trained for the TF or RBP binding prediction problem and a phylogenetic tree, *ψ*, where each node represents the real-valued score assigned by a base predictor to the corresponding orthologous sequence. A simple way to combine the predictions would be to take a weighted average. However, this ignores the dependencies modeled by the tree structure and a smaller number of strong predictions can get undermined by a majority of weak predictions. A better approach could be to utilize a probabilistic graphical model view of combining the scores.

Consider a phylogenetic tree, *ψ*, with *n* nodes, where index 1 is the root, *s_i_* denotes the base model score assigned to node *i*, and *e_ij_* is the evolutionary distance between parent *i* and descendant *j*. Let the label of the root species be *Y*. Then the probability of *Y *=* y* given the set of prediction scores is:

(1) $$\begin{array}{c} P[Y=y\;|\;s_{1},s_{2},\ldots ,s_{n}]\propto P[s_{1},s_{2},\ldots ,s_{n}|\;Y=y]\cdot P[Y=y]\\ =P[s_{1}|Y=y]\cdot \prod_{\left(p,c)\in \mathrm{edges}(\psi \right)}P[s_{c}|s_{p},Y=y,e_{p,c}] \end{array},$$

where *p*, *c* are parent–descendant pairs and $e_{p,c}$ is the evolutionary distance between them in *ψ*.

The final combined score to predict *Y* is the log-likelihood ratio of Equation 1 with *Y *=* *1 and *Y *=* *0, where 1 and 0 denotes positive and negative labels, respectively:

(2) $$\text{PhyloPGM}\_\text{Score}=\text{log}(\frac{P[Y=1\;|\;s_{1},s_{2},\ldots ,s_{n}]}{P[Y=0\;|\;s_{1},s_{2},\ldots ,s_{n}]}),$$

 

(3) $$=\text{log}(\frac{P[s_{1}|Y=1]}{P[s_{1}|Y=0]})+\sum_{\left(p,c)\;\in \;\mathrm{edges}(\psi \right)}\;\text{log}\;(\frac{P[s_{c}|s_{p},Y=1,e_{p,c}]}{P[s_{c}|s_{p},Y=0,e_{p,c}]}),$$

 

(4) $$\propto \;\text{log}\;\frac{P[s_{1}|Y=1]}{P[s_{1}|Y=0]}+\alpha \cdot \sum_{\left(p,c)\;\in \;\mathrm{edges}(\psi \right)}\;\text{log}\;\frac{P[s_{c}|s_{p},Y=1,e_{p,c}]}{P[s_{c}|s_{p},Y=0,e_{p,c}]},$$

where *α* is a model hyper-parameter to balance the effect of likelihood ratio of non-root species.

The conditional probabilities ($P[s_{c}|s_{p},Y=y,e_{p,c}]$) of the base model score on a descendant species given the parent score, label and the evolutionary distance is difficult to compute. We estimate the conditional probabilities of scores on root node and over each edge empirically from the scores in the training dataset, *T*. In order to empirically estimate the conditional probabilities, the base prediction scores, which are assumed to be between 0 and 1, are discretized by rounding to the first decimal place and binned in 12 bins (11 bins corresponding to 0, 0.1, 0.2,…, 0.9, 1.0 and one extra bin for the missing values as orthologous region in a particular species may not be present). The required probabilities in the Equation 4 are estimated as,

$$\begin{array}{c} P[s_{1}|Y=y]=\frac{\sum_{i\in T}1_{s_{1}^{i}=s_{1}\wedge l(i)=y}+\epsilon }{\sum_{i\in T}1_{l(i)=y}+12\epsilon }\\ P[s_{c}|s_{p},Y=y]=\frac{\sum_{i\in T}\;1_{s_{c}^{i}=s_{c}\wedge s_{p}^{i}=s_{p}\wedge l(i)=y}+\epsilon }{\sum_{i\in T}\;1_{s_{p}^{i}=s_{p}\wedge l(i)=y}+12\epsilon } \end{array},$$

where *T* is training data, $s_{1}^{i}$ is base model score on human in *i*th example, *l*(*i*) is label of *i*th example, *ϵ* is a pseudo count set to *ϵ *= 1 (empirically chosen from [0.01, 0.1, 1, 10])

It should be noted that for a given example, *s_c_* or *s_p_* may be missing due to the absence of orthologous regions in the corresponding species. The missing values are ignored in such cases. Furthermore, the multinomial distribution used to compute the log-likelihood ratio implicitly involves the evolutionary distance between the descendant and parent. In this study, we use the phylogenetic tree available from the UCSC genome browser (https://hgdownload.soe.ucsc.edu/goldenPath/hg38/multiz100way/) and rerooted the tree so that the human species becomes the root node. We empirically selected α=0.1 from [0.001,0.01,0.1,1,10,100,1000].

### 2.7 PhyloStackNN approach

The goal of the stacking approach, PhyloStackNN, is to test the importance of the explicit use of the phylogenetic tree in the PhyloPGM approach. PhyloStackNN is a simple multi-layer perceptron that takes base predictor scores $s_{1},s_{2},\ldots ,s_{n}$ as input and is trained to predict the label. It is trained on the same train/test split as PhyloPGM. The MLP architecture is chosen from the hyper-parameter search over {‘hidden_layer_sizes’: [(32,), (100,), (64, 32)], ‘$\ell_{2}$ penalty’: [0.1, 1, 10]} using 10-fold cross-validation.

## 3 Results

In this section, we present and evaluate the results obtained using PhyloPGM for the tasks of binary TF and RBP occupancy prediction. In both cases, given a (1000 bps) DNA or (101 nts) RNA sequence, the goal is to predict whether a given TF or RBP would bind this sequence in a given cell type. The input sequence is much longer than the putative binding site itself, which provides important sequence context (e.g. for the presence of binding sites for co-factors, or structural RNA elements) to the base predictor.

### 3.1 PhyloPGM improves predictors’ performance

We first applied PhyoPGM to the task of TF occupancy prediction, using FactorNet (Quang and Xie, 2019) as base predictor. FactorNet is a recently developed hybrid of convolutional and recurrent neural network architectures, which performed particularly well on a recent ENCODE-DREAM challenge (Kundaje *et al.*, 2021). We used a set of 13 ChIP-Seq datasets, obtained from the ENCODE-DREAM website (https://www.synapse.org/#! Synapse: syn6131484/wiki/402026). The datasets originate from four different cell types and contain 56 700 to 423 218 positive examples and 50 356 411 to 51 164 150 negative examples (see Section 2.1).

**Fig. 2.**
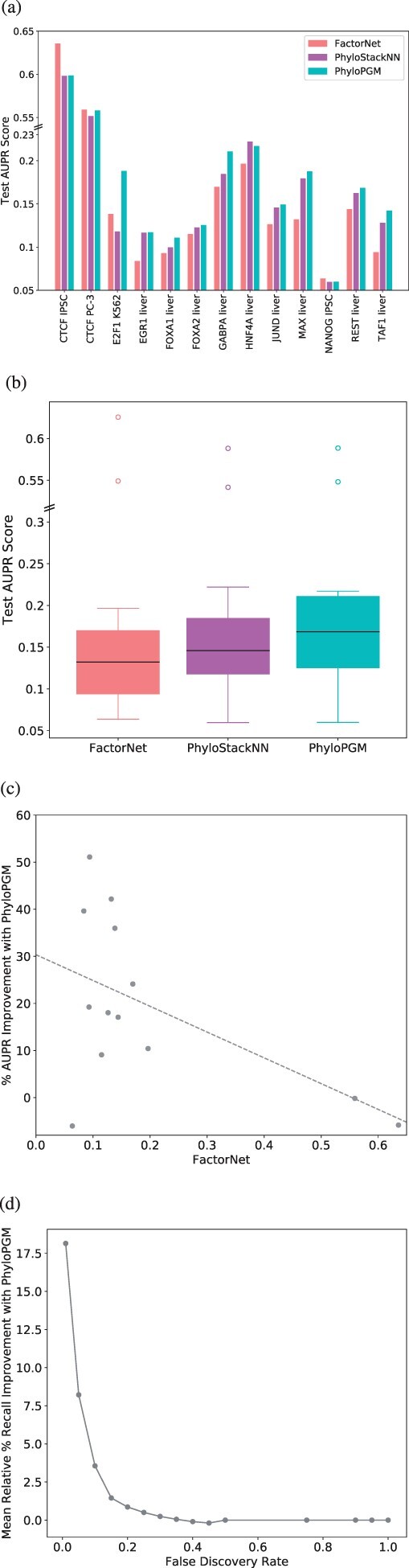
Model comparison for TFBS prediction problem. (**a**) Test AUPR scores of FactorNet, PhyloStackNN and PhyloPGM over 13 ChIP-Seq datasets. (**b**) Distribution of test AUPR. (**c**) Test AUPR improvement percentage of PhyloPGM over FactorNet. (**d**) Mean relative percentage improvement of PhyloPGM test recall score over FactorNet for different false discovery rate thresholds

The performance of the predictors is evaluated on the test set provided by ENCODE-DREAM (Kundaje *et al.*, 2021), using the area under the precision-recall curve (AUPR), which better reflects the true predictor’s performance with imbalanced datasets, compared to the more traditional AUC score. Overall, PhyloPGM improves the AUPR scores of the FactorNet models by approximately 30% (FactorNet median test AUPR: 0.13, PhyloPGM median test AUPR: 0.17; Wilcoxon signed-rank test *P*-value: 0.019) (see Fig. 2a and b). It should be noted that the FactorNet results we report are different from Quang and Xie (2019) because we use only sequence information while Quang and Xie (2019) use both sequence and non-sequence information. We also evaluated another approach, called PhyloStackNN, which uses a neural network to learn to optimally combine the base prediction scores but without prior knowledge of the phylogenetic tree (see Section 2.7). PhyloPGM outperforms PhyloStackNN by a smaller margin (PhyloStackNN median test AUPR: 0.15; Wilcoxon signed-rank test *P*-value: 0.0058), which shows that utilizing the phylogenetic tree to combine the orthologous scores is indeed helpful. Notably, PhyloPGM seems particularly effective at improving prediction accuracy in liver, and less so in IPSCs.

**Fig. 3.**
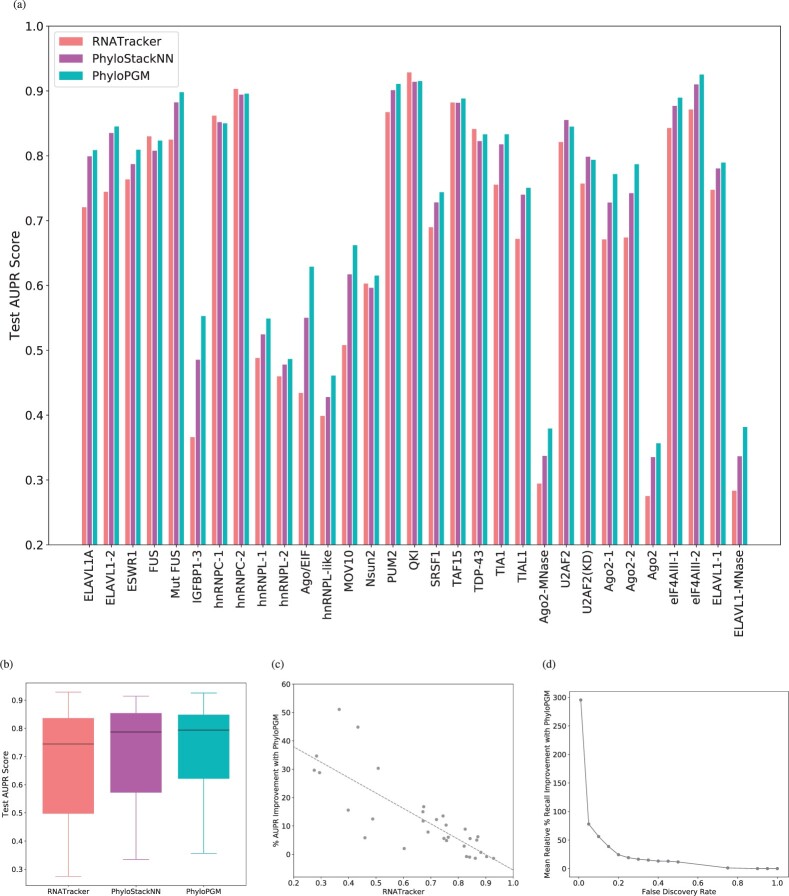
Model comparison for RNA binding prediction problem. (**a**) Test AUPR scores of RNATracker, PhyloStackNN and PhyloPGM over 31 CLIP-Seq datasets. (**b**) Distribution of test AUPR. (**c**) Test AUPR improvement percentage of PhyloPGM over RNATracker. (**d**) Mean relative percentage improvement of PhyloPGM test recall score over RNATracker for different false discovery rate thresholds

We repeated a similar evaluation for the RBP occupancy prediction task based on 31 CLIP-Seq datasets from Stražar *et al.* (2016). These data were collected in HEK293, HeLa and U266 cell types and contain 3283 to 6000 positive examples and 23 672 to 26 214 negative examples (see Section 2.2). Here, we used RNATracker (Yan *et al.*, 2019), a hybrid of convolutional and recurrent neural networks, as such architectures have shown remarkable prediction accuracy with sequence function prediction tasks (Pan *et al.*, 2018; Quang and Xie, 2019). Again, we find that PhyloPGM outperforms the base predictor (RNATracker median test AUPR: 0.74, PhyloPGM median test AUPR: 0.793; Wilcoxon signed-rank test *P*-value: $8.65\times 10^{-6}$) (see Fig. 3a and b). PhyloPGM improves upon the base model in 26 of the 31 datasets; for the remaining 5 datasets (FUS, hnRNPC-1/2, QKI, TDP-43), the AUPR scores differ by <1%. Similar to the TFBS prediction problem, we find that the PhyloPGM appraoch performs better than the PhyloStackNN approach where the phylogenetic relationship is not used (PhyloStackNN median test AUPR: 0.787; Wilcoxon signed-rank test *P*-value: $6.57\times 10^{-6}$).

### 3.2 Improvement to the Recall score

In general, TF and RBP binding predictors suffer from high false discovery rates, due to the imbalanced classes of positive and negative examples. Thus, apart from AUPR scores, such models should also be evaluated on the recall score at different false discovery rates (FDRs). Figures 2d and 3d report the relative percentage improvement in the mean recall scores of PhyloPGM over FactorNet and RNATracker at different FDR thresholds. We find that PhyloPGM yields particularly large gains in recall at low FDR ranges (FDR <0.1), which is the range of particular interest for genome-wide applications. The relative improvement in the recall score at 1% FDR is ∼18% with PhyloPGM over FactorNet in the ChIP-Seq datasets and ∼300% over RNATracker in the CLIP-Seq datasets.

### 3.3 PhyloPGM most significantly improves weaker models

One of the main PhyloPGM design motivations is to exploit orthologous data in order to make correct predictions for examples that are otherwise difficult to classify using only the base predictor. We observe that the degree of improvement with PhyloPGM over FactorNet and RNATracker is more substantial for weaker base models, i.e. for datasets where the base models obtain a low AUPR scores (see Figs 2c and 3c). This confirms our belief that the information from orthologous/ancestral sequences is particularly beneficial for hard-to-predict TFs and RBPs.

### 3.4 Contribution of each phylogenetic tree branches

The PhyloPGM score is essentially a sum of log-likelihood ratios over the branches of the tree, with the change in prediction score observed along each branch contributing to nudging the final prediction towards the positive or negative class. Hence it is meaningful to investigate which branch of the tree contributes most to the boost of prediction accuracy obtained by PhyloPGM. To this end, we computed, for each datasets and each branch in the tree, the mean difference of the branch log-likelihood ratio of positive and negative examples (see Supplementary Figs S1 and S3). The branches most beneficial to the PhyloPGM predictions are those where this difference is largest. Notably, nearly all branches are at least minimally useful for all datasets, justifying the use of the full phylogenetic tree. However, the extent of branch-specific signals are beneficial varies significantly. For TF occupancy prediction tasks (Supplementary Fig. S1), branches closest to human are generally the most predictive value. This is particularly true for CTCF and Nanog, which also happen to be those datasets obtained from IPSC cell type, and for which PhyloPGM underperforms. We hypothesize that many of the human binding sites for these proteins may have arisen recently during primate evolution, as suggested by Ni *et al.* (2012) and Scerbo *et al.* (2014). On the contrary, transcription factors such as E2F1, GABPA and TAF1 (all assayed in liver) display a high level of branch informativeness across much of the mammalian tree. This suggests a lower turnover of regulatory regions for those TFs.

To investigate the role of conservation in more details, we compared the percentage improvement from PhyloPGM in AUPR with the mean of PhastCons scores in the bound examples for each dataset (see Supplementary Figs S2 and S4). We observe that the amount of improvement in AUPR is highly correlated with the PhastCons scores. Moreover, the majority of CLIP-seq data have higher PhastCons scores than the ChIP-Seq data, which is expected due to RNA binding sites being generally more conserved than the TFBSs (Payne *et al.*, 2018). Therefore, PhyloPGM seems to be more effective in boosting the binding prediction accuracy of TFs and RBPs whose binding sites are more conserved. The major exception to this trend is NANOG in IPSC cell type, may be due to the absence of binding sites in the orthologous regions (Scerbo *et al.*, 2014).

### 3.5 PhyloPGM helps identifying disease-causing human non-coding variants

ChIP-Seq and CLIP-Seq experiments are limited to the question of whether a given protein binds a certain genomic region or not but do not reveal information on the functional consequences of this interaction. Indeed, many binding events appear to have no or only limited consequences on gene expression (Barakat *et al.*, 2018; Vanhille *et al.*, 2015), and hence tend to be evolutionarily neutral. Because PhyloPGM indirectly measures the level of selective pressure to maintain the binding potential of a region for a given TF/RBP, it stands to reason that regions with high PhyloPGM scores not only have a higher chance of being bound but also that this binding event is more likely to be of functional consequences.

To test this hypothesis, we used a variety of external data sources to identify binding events that are more likely to be of functional consequences, including: (i) the non-coding portion of the ClinVar database (Landrum *et al.*, 2016), which human mutations associated to diseases; (ii) the non-coding human variants linked to phenotypic consequences through several publications (Biggs *et al.*, 2020); (iii) the list of deleterious non-coding variants identified through machine learning and other computational techniques (Wells *et al.*, 2019). Regions of the human genome bound by a TF/RBP and overlapping at least of those datasets are deemed more likely to harbor functional binding events and are called *putatively functional*.

We then measured, for each TF/RBP, the extent to which the bound regions that are assigned the highest PhyloPGM scores (top 30%) overlap the set of putatively functional sites. The same procedure was applied to the top regions ranked based on the base predictor (FactorNet or RNATracker) or a simpler measure of sequence conservation (PhastCons).

**Fig. 4.**
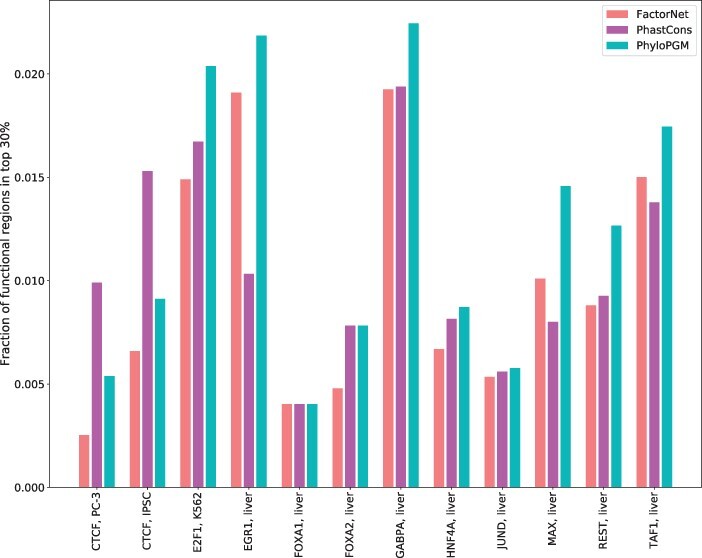
Fraction of (top 30%) high-scoring regions from FactorNet, PhastCons and PhyloPGM that overlap with the putative functional TFBSs in the ChIP-Seq data used in this study

**Fig. 5.**
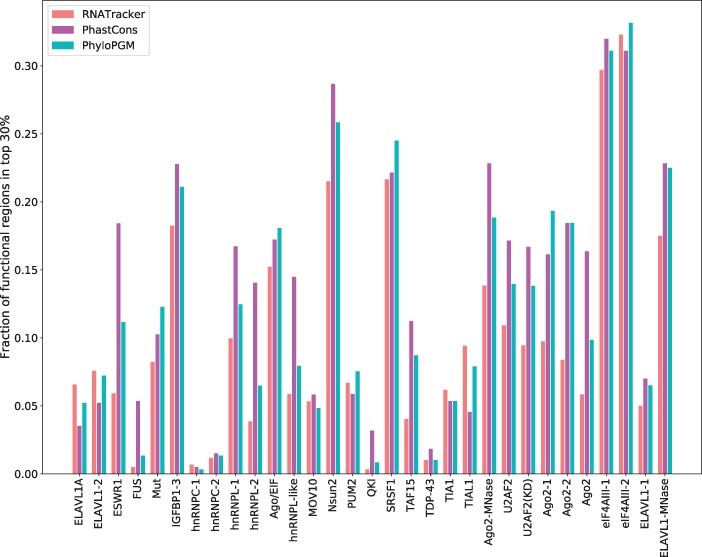
Fraction of (top 30%) high-scoring regions from RNATracker, PhastCons and PhyloPGM that overlap with the putative functional RBP binding sites in the CLIP-Seq data used in this study

Figure 4 shows that for 11 of the 12 TF datasets, high-scoring putatively functional TF binding sites are more commonly found within high-scoring PhyloPGM sites e.g. (E2F1, K562), (EGR1, liver) and (GABPA, liver). Similarly, 24 out of 31 CLIP-Seq datasets have more overlapping putative functional RBP binding sites with regions assigned a high score by PhyloPGM than the base model e.g. SRSF1, Nsun2 and TAF15 (see Fig. 5). This is an interesting benefit of PhyloPGM because PhyloPGM not only boost the base model performance but is also more predictive of the functional aspects.

## 4 Discussion

We present PhyloPGM, an aggregation approach to boost the prediction accuracy of a previously trained TF or RBP binding predictor on human sequences. The accuracy of a base model trained on human sequences may change in other species (Villar *et al.*, 2014) and the performance of a base model on other species is difficult (or impossible in the case of an ancestor) to evaluate due to lack of data. If binding affinity of a given TF changes in other species then the likelihood ratio corresponding to those species in Equation 4 should be closer to zero. Thus, the change in the binding affinity of TFs in other species shall reduce the power of PhyloPGM but will not invalidate PhyloPGM results.

We have shown that PhyloPGM significantly improves the median AUPR scores of FactorNet and RNATracker models trained on human sequences by more than 4% in 13 ChIP-Seq datasets and 5% in 31 CLIP-Seq datasets. PhyloPGM, in principle, is designed to improve the prediction accuracy of the labeled examples that are difficult to classify i.e. the examples that lie closer to the decision boundary. Indeed, our analysis shows that the log-likelihood ratios between parents and descendants in the orthologous set improve the prediction quality of such examples. The most significant improvements in the AUPR score of PhyloPGM are observed on datasets where FactorNet or RNATracker have performed relatively poorly. Moreover, we show that the explicit use of the phylogenetic tree provides significant gain for PhyloPGM over PhyloStackNN, which combines the orthologous prediction scores with a neural network without taking into account of the phylogenetic relationship. Additionally, PhyloPGM is shown to have better recall scores at lower false discovery rates than the base models in both ChIP-Seq and CLIP-Seq datasets.

We find that the datasets showing more improvement with PhyloPGM over base models have relatively higher PhastCons scores i.e. the sequences are more conserved. We observe that PhyloPGM improves the base model relatively more in CLIP-Seq data compared to ChIP-Seq data. The RNA binding sites are mostly observed in the 3′ UTR region, which are generally more conserved than transcriptional regulatory regions. This may explain the comparatively better performance of PhyloPGM in CLIP-Seq data. Furthermore, binding site turnover may affect transcriptional regulatory regions more than 3′ UTRs, which may cause loss of or larger shifting of binding sites in the orthologs.

The comparison of branches in the phylogenetic tree in terms of the impact of the likelihood ratio on PhyloPGM shows that the branches that are farther from human are relatively more useful. However, the branch likelihood ratio seems to be less/not useful after a certain distance from the human, which may indicate loss of binding sites in such orthologs. In the similar direction, we should explore other phylogenetic relationships such as the effect of using only a subset of species on the PhyloPGM accuracy, the relationship between the regulatory function associated with a sequence and its conservation across different species. More of such investigations should allow us to identify important evolutionary changes that had an impact on regulatory regions. Additionally, this should open up the possibility of using PhyloPGM as a potential comparative genomics tool that can be applied in many other related areas e.g. therapeutic approaches, studying the evolution of regulatory activities and other functions related to biological sequences. Furthermore, the application of PhyloPGM on a subset of useful species rather than the entire orthologs shall reduce the computational overhead of running PhyloPGM with either loss or gain in the accuracy.

An important observation from the analysis with the ClinVar datasets is that PhyloPGM is more predictive of the human genomic regions where mutations are linked to diseases. One aspect of results with ClinVar datasets is that PhyloPGM is capable of identifying deleterious regions. Moreover, one can compare base model predictions on a reference genome and an individual genome to filter the regions with significant prediction differences. Then, PhyloPGM can be applied on these selected regions of an individual genome to detect regions with any concerned mutations. The other aspect of results with ClinVar datasets is that the regions where mutations are linked to diseases could be considered as functional, in the sense that mutations in such regions could affect the fitness of species. Such regions should be associated with some regulatory activities. Additionally, the ChIP-seq and CLIP-seq experiments are not free from noise (e.g. false TF or RBP binding sites, inconsequential binding etc.) (Barakat *et al.*, 2018; König *et al.*, 2012; Moore *et al.*, 2014; Ule *et al.*, 2005; Vanhille *et al.*, 2015). It may also be the case that such wet-lab experiments identify a genomic location as a potential binding site for a TF or RBP, and yet, a TF or RBP binding to such location has no impact on any regulatory activity. Improving the wet-lab experiment data with more functional regions (i.e. identified binding sites have some role in a regulatory activity) may result into further improvement in the accuracy with PhyloPGM. The improved PhyloPGM scores can further be used to identify regions associated with regulatory activities. Contrastingly, the improvement in accuracy with PhyloPGM will be biased towards more conserved binding sites. Therefore, PhyloPGM is not suitable for the study of gene regulation that are associated with less conserved regions e.g. regulatory study of a gene responsible for human brain development that may not be in other species. However, these specific cases are very rare and PhyloPGM should be beneficial in the majority of gene regulation study.

At present, PhyloPGM is formulated for solving a binary classification task, and it may be potentially extended to multi-classification tasks (e.g. in an one-versus-all setting). This should allow PhyloPGM to be applicable to other sequence function prediction tasks that involves more than one labels, for example, protein function prediction (Kulmanov and Hoehndorf, 2020) and mRNA subcellular localization (Yan *et al.*, 2019). PhyloPGM is inherently designed for classification tasks and will require modifications in order to be applicable to regression-based sequence function prediction tasks e.g. predicting gene expression value from a sequence. The discretization of regression values may allow the application of PhyloPGM in regression tasks. Furthermore, the use of beta distribution and conditional multivariate distribution in place of multinomial distribution may allow to better fit the log-likelihood ratio of the branches in PhyloPGM pipeline. Although many sequence function prediction tasks have computational models and datasets (e.g. Kulmanov and Hoehndorf, 2020; Leclercq *et al.*, 2017; Yan *et al.*, 2019), applying PhyloPGM to them will require necessary adjustments w.r.t. the base predictors and datasets. The datasets size and base predictor forms vary from one sequence function prediction tasks to another. Furthermore, the improvement in accuracy and evolutionary insights from PhyloPGM for a given sequence function prediction task depends on the base predictor and the datasets (s.t. sequence function is maintained during evolution).

## Supplementary Material

btac259_Supplementary_Materials
