# Supplementary material for: PhyloPGM: boosting regulatory function prediction accuracy using evolutionary information
Source: Bioinformatics. 2022 Jun 27;38(Suppl 1):i299–306. doi: 10.1093/bioinformatics/btac259 (PMC9235490; doi:10.1093/bioinformatics/btac259)
Supplement: btac259_Supplementary_Materials [file btac259_supplementary_materials.zip › btac259_Supplementary_Materials/Ahsan.80.sup.1.pdf]

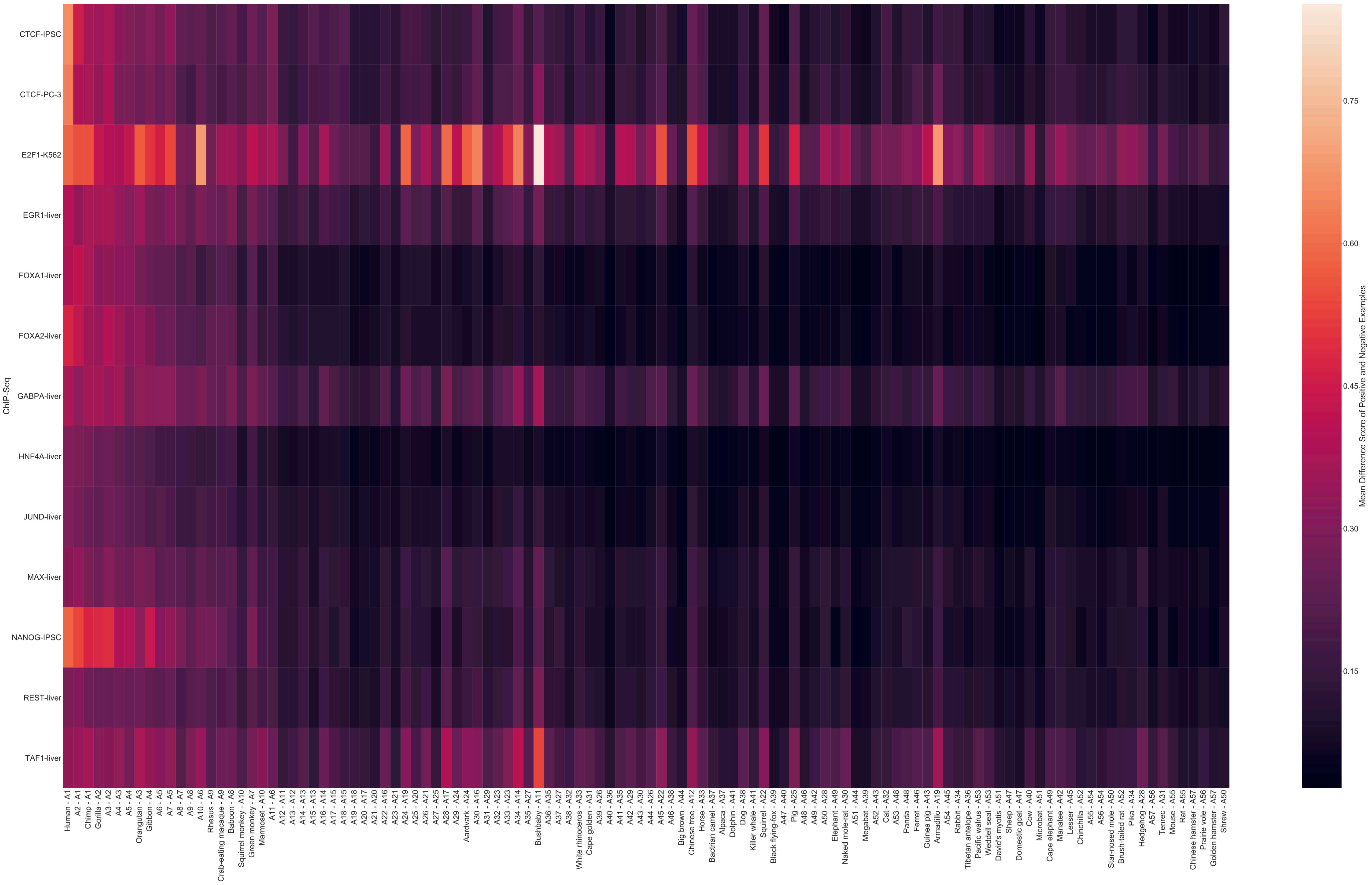

Figure S1. PhyloPGM analysis with TFBS prediction problem. Each cell represents difference of mean of branch likelihood ratio of positive and negative examples for a branch of the phylogenetic tree in a Chip- Seq experiment. The examples are represented as positive or negative based on the human orthologue. The branch likelihood ratio is computed from the FactorNet scores on the orthologous examples. The columns are sorted w.r.t evolutionary distance of the branch from human. Each column is named as species followed by its direct parent. The species Ai denotes ancestral species, where i indicates the evolutionary distance from human (i=1 is closest to human).
