# Supplementary material for: PhyloPGM: boosting regulatory function prediction accuracy using evolutionary information
Source: Bioinformatics. 2022 Jun 27;38(Suppl 1):i299–306. doi: 10.1093/bioinformatics/btac259 (PMC9235490; doi:10.1093/bioinformatics/btac259)
Supplement: btac259_Supplementary_Materials [file btac259_supplementary_materials.zip › btac259_Supplementary_Materials/Ahsan.80.sup.4.pdf]

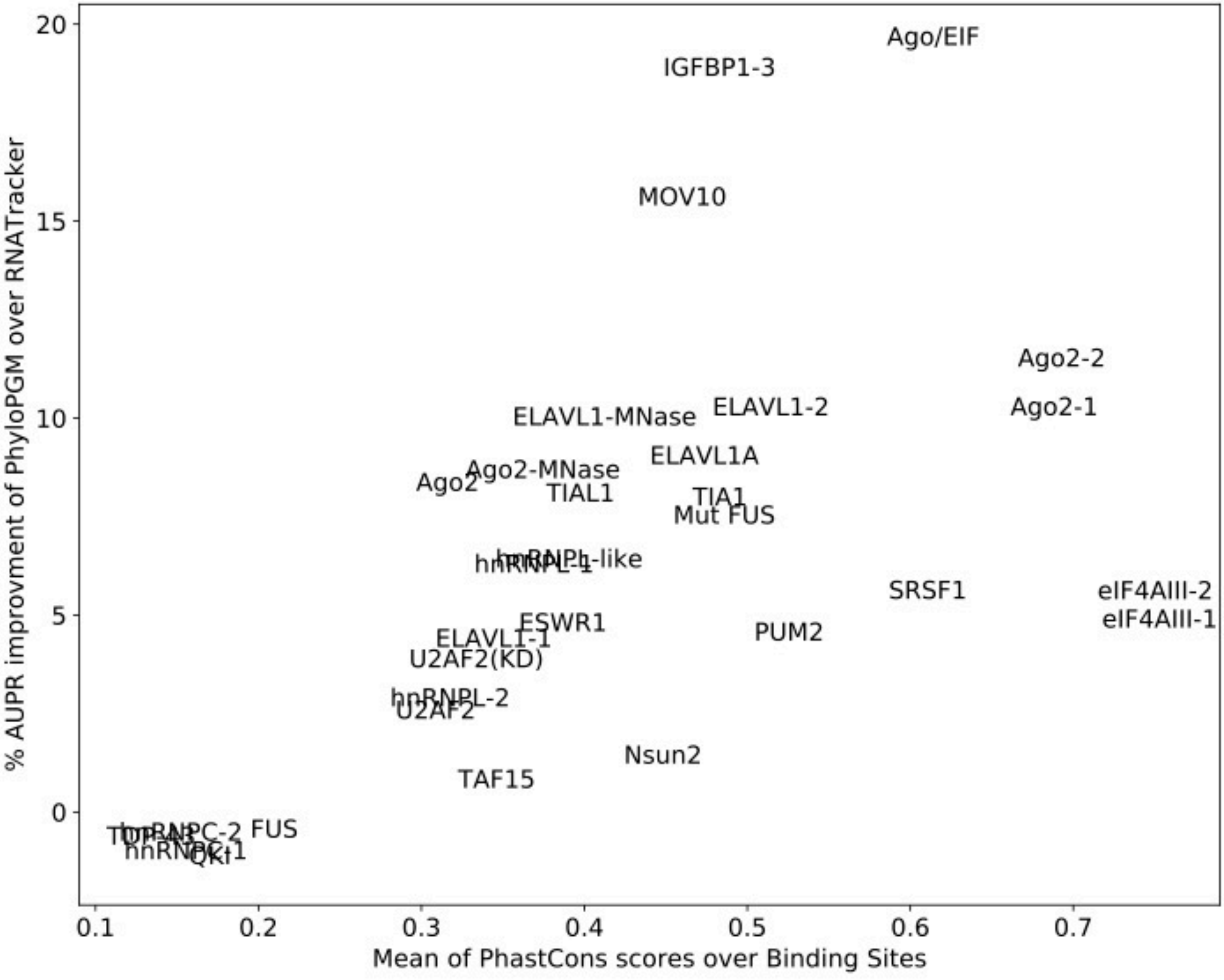

Figure S4. Comparison of improvement with PhyloPGM over RNATracker and conservation score. The datasets with relatively high conservation scores showed relatively high amount of improvement with PhyloPGM.
